# Association of High LAT1 Expression with Poor Prognosis and Recurrence in Colorectal Cancer Patients Treated with Oxaliplatin-Based Adjuvant Chemotherapy

**DOI:** 10.3390/ijms24032604

**Published:** 2023-01-30

**Authors:** Yuta Shibasaki, Takehiko Yokobori, Makoto Sohda, Ikuma Shioi, Naoya Ozawa, Chika Komine, Kunihiko Suga, Nobuhiro Nakazawa, Katsuya Osone, Takuya Shiraishi, Takuhisa Okada, Akihiko Sano, Makoto Sakai, Hiroomi Ogawa, Kyoichi Kaira, Ken Shirabe, Hiroshi Saeki

**Affiliations:** 1 Department of General Surgical Science, Graduate School of Medicine, Gunma University, Maebashi 371-8510, Japan; 2 Division of Integrated Oncology Research, Gunma University, Initiative for Advanced Research (GIAR), Maebashi 371-8511, Japan; 3 Department of Respiratory Medicine, Comprehensive Cancer Center, International Medical Center, Saitama University Hospital, Hidaka 350-1298, Japan

**Keywords:** L-type amino acid transporter-1, cancer aggressiveness, prognostic marker, chemosensitivity marker

## Abstract

The mammalian target of rapamycin (mTOR) is often activated in several cancers. We focused on two mTOR regulatory mechanisms: oxaliplatin-induced mTOR signaling and L-type amino acid transporter 1 (LAT1)-induced mTOR activation. High LAT1 expression in several cancers is associated with mTOR activation and resistance to chemotherapy. However, the significance of LAT1 has not yet been elucidated in colorectal cancer (CRC) patients treated with post-operative adjuvant chemotherapy. Immunohistochemistry was conducted to examine the significance of membrane LAT1 expression in 98 CRC patients who received adjuvant chemotherapy, including oxaliplatin. In vitro analysis was performed using CRC cell lines to determine the effects of LAT1 suppression on proliferation, oxaliplatin sensitivity, and mTOR signaling. LAT1 expression was associated with cancer aggressiveness and poor prognosis in 98 CRC patients treated with adjuvant chemotherapy. We found that positive LAT1 expression correlated with shorter survival in 43 patients treated with the capecitabine-plus-oxaliplatin (CAPOX) regimen. LAT1 suppression in CRC cells inhibited the proliferation potency and oxaliplatin-induced activation of mTOR signaling, and improved oxaliplatin sensitivity. LAT1 evaluation before adjuvant treatment may therefore be a sensitive marker for oxaliplatin-based regimens. Moreover, LAT1 may be a promising target for patients with refractory CRC.

## 1. Introduction

Colorectal cancer (CRC) is a commonly diagnosed cancer and a leading cause of cancer-related deaths worldwide [1,2]. For patients with operable CRC, radical surgical resection is an essential curative treatment, and post-operative adjuvant chemotherapy has been reported to improve survival and prevent disease recurrence [3,4,5]. More than half of post-radical resection CRC patients without adjuvant chemotherapy do not relapse; however, some patients relapse despite adjuvant chemotherapy [6,7,8]. Therefore, there is a need to develop methods to select patients at high risk of recurrence, which existing clinicopathologic factors cannot predict, and to overcome resistance to adjuvant chemotherapy.

Activation of the mammalian target of rapamycin (mTOR) has often been observed in several types of cancer [9,10]. In addition, mTOR is involved in tumor progression and resistance to therapy in many carcinomas, including CRC, through the regulation of amino acid metabolism and protein synthesis. Therefore, cancer treatment strategies that inhibit mTOR have attracted much attention and achieved clinical successes [11,12,13]. Although various factors activate mTOR signaling, this study focused on two mTOR regulatory mechanisms: anti-cancer drug-induced mTOR signaling and L-type amino acid transporter 1 (LAT1) (also referred to as SLC7A5)-induced mTOR activation [14]. In CRC, adjuvant chemotherapy, including oxaliplatin, is often administered to eradicate minimal residual disease and prevent the recurrence of CRC after radical resection [4,5]. However, oxaliplatin in adjuvant chemotherapy regimens has unexpectedly been reported to activate mTOR signaling in response to chemotherapy resistance, suggesting a biological response of cancer cells to the cell-killing mechanism of anti-cancer drugs [15]. Moreover, several researchers have previously reported that high expression of LAT1 in several resected cancer samples, including CRC, is associated not only with mTOR activation, but also with cancer metastasis, recurrence, and poor prognosis [16,17,18,19,20,21,22,23].

It has been reported that LAT1 may regulate amino acid uptake and metabolism with regard to cancer aggressiveness and therapeutic resistance [17,24,25]. We previously reported a vital relationship between LAT1 and chemosensitivity in pancreatic cancer, indicating that high expression of tumoral LAT1 in pancreatic cancer patients treated with adjuvant chemotherapy was associated with shorter survival, compared to cases with low LAT1 expression. We also found that patients who were non-responder cases to systemic chemotherapy after recurrence of pancreatic cancer had a high incidence of LAT1, compared to responders. Our in vitro analysis validated that the chemosensitivity of LAT1-suppressed pancreatic cancer cells was higher than that of control cells, which was consistent with the expected LAT1 function in clinical pancreatic cancer [22]. The crucial finding that cancerous LAT1 accumulation is associated with shorter survival after adjuvant chemotherapy has also been validated in clinical gastric cancer specimens [26]. These studies indicate the potential of LAT1 as a valuable prognostic marker for cancer patients undergoing radical resection and adjuvant therapy, and as a therapeutic target against chemoresistant refractory cancers. However, the significance of LAT1 in tumor aggressiveness, prognosis, and mTOR signaling has not yet been elucidated in CRC patients treated with post-operative adjuvant chemotherapy, including oxaliplatin.

This study aimed to clarify the clinical significance and prognostic value of LAT1 expression in 98 patients with CRC who received adjuvant therapies, including oxaliplatin-based regimens. Moreover, we performed an in vitro analysis using CRC cell lines to determine the effects of LAT1 suppression on proliferation, oxaliplatin sensitivity, and mTOR signaling activity related to chemoresistance.

## 2. Results

### 2.1. Immunohistochemical Staining of LAT1 in Clinical CRC Specimens

**Figure 1:**
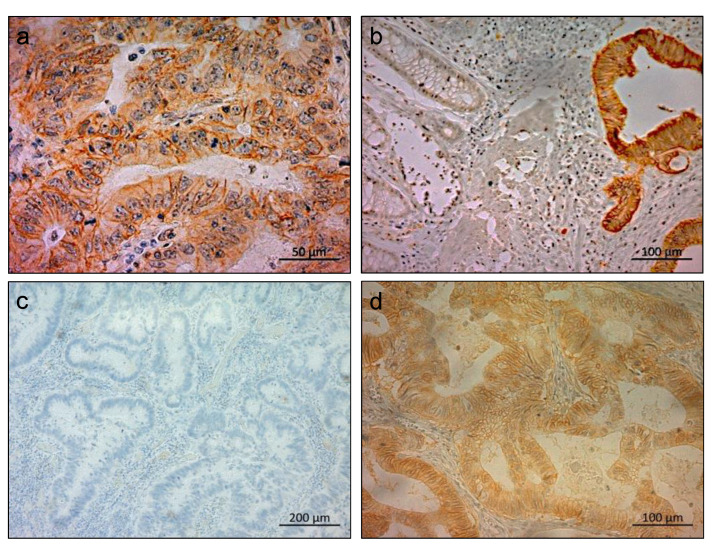
Immunohistochemical staining of LAT1 in clinical CRC samples. (**a**) Representative immunohistochemical staining of membrane LAT1 expression in cancerous areas of CRC tissues (scale bar, 50 μm) (**b**) Representative immunohistochemical staining of membrane LAT1 in the cancer part and non-cancerous colon mucosa (scale bar, 100 μm). Stronger LAT1 expression was observed in cancer tissues compared to normal colon mucosa. (**c**) Representative section of a CRC tissue with LAT1-negative expression. (**d**) Representative section of a CRC tissue with LAT1 positive expression.

Within the clinical samples, LAT1 was mainly expressed in the membranes of CRC cells (Figure 1a). Higher LAT1-expression levels were detected in cancerous tissues compared to normal colon mucosa (Figure 1b). Of the 98 CRC specimens, 45 (45.9%) and 53 (54.1%) were classified as LAT1 negative (Figure 1c) and LAT1 positive groups (Figure 1d), respectively.

### 2.2. Clinicopathological Significance of LAT1 Expression in Patients with CRC

Table 1 shows the relationship between LAT1 positivity and various clinicopathological factors in 98 patients with CRC. The LAT1-positive group was significantly associated with the progression of lymph node metastasis (*p* = 0.02), lymphatic invasion (*p* = 0.044), and recurrence (*p* = 0.007) compared to the negative group (Table 1).

**Figure 2:**
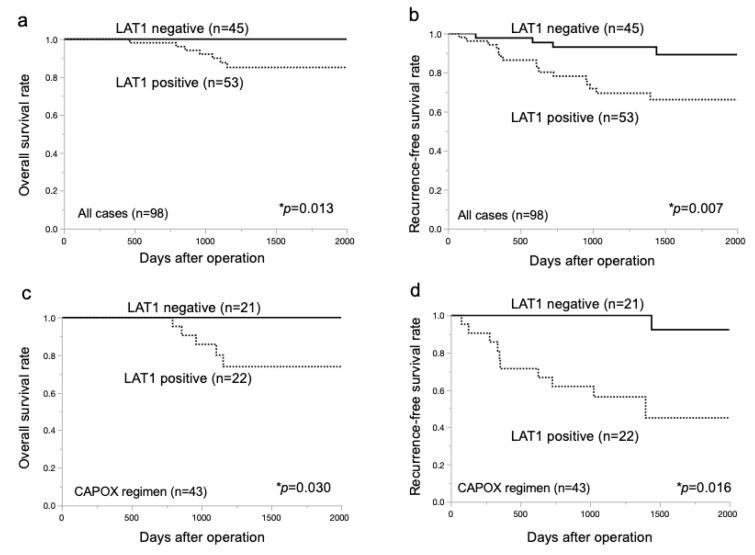
Kaplan–Meier survival curves of patients with CRC according to LAT1 expression. (**a**,**b**) Kaplan–Meier analysis of overall and recurrence-free survival in 98 CRC patients treated by adjuvant chemotherapy (*p* = 0.013 and *p* = 0.007, respectively). The analyses were based on LAT1 expression. (**c**,**d**) Kaplan–Meier analyses of overall survival and recurrence-free survival in 43 CRC patients treated with CAPOX regimen, including oxaliplatin, according to LAT1 expression (*p* = 0.03 and *p* = 0.016, respectively).

Kaplan–Meier analysis revealed significantly lower overall survival and recurrence-free survival rates in the LAT1-positive group than in the negative group (*p* = 0.013, Figure 2a, and *p* = 0.007, Figure 2b, respectively). Among the 43 patients treated with the CAPOX regimen, the LAT1-positive group (*n* = 22) was significantly associated with shorter overall survival and recurrence-free survival than the negative group (*n* = 21) (*p* = 0.03, Figure 2c, and *p* = 0.016, Figure 2d, respectively). However, the prognostic value of LAT1 was not observed in other regimens (Supplementary Figure S1).

### 2.3. Prognostic Significance of LAT1 Expression in Patients with CRC

Multivariate analysis showed that the LAT1-positive group and T factor were independently associated with recurrence in CRC patients treated with adjuvant chemotherapy (hazard ratio (HR), 3.99; 95% confidence interval (CI), 1.327–11.99; *p* = 0.014, HR, 3.28; 95% CI, 1.309–8.234; *p* = 0.011, respectively (Table 2). In addition, among the CAPOX regimen group (*n* = 43), multivariate analysis showed that LAT1 positivity was an independent predictor of recurrence (HR, 12.53; 95% CI 1.549–101.4; *p* = 0.018) (Supplementary Table S1).

### 2.4. Functional Analysis of LAT1 in CRC Cell Lines

**Figure 3:**
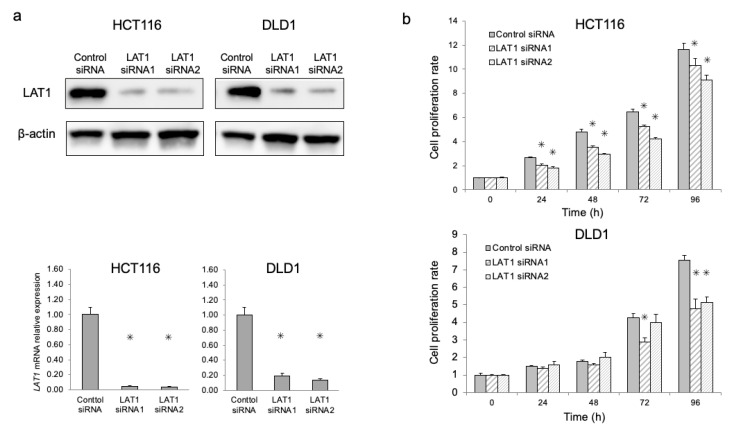
Functional analysis of LAT1 in human CRC cell lines. (**a**) LAT1 suppression was evaluated in HCT116 and DLD1 cells treated with LAT1 siRNAs by Western blotting and qRT-PCR. β-actin was used to normalize the loading control for the Western blot. (**b**) The proliferation of CRC cells after LAT1 siRNA treatment was evaluated using a Cell Counting Kit-8 kit. * *p* < 0.05.

HCT116 and DLD1 cell lines were used for subsequent knockdown experiments to analyze the functional significance of LAT1 in cell proliferation and oxaliplatin sensitivity assays. We used siRNAs to silence LAT1 expression, and validated LAT1 suppression by Western blotting and qRT-PCR (*p* < 0.05, Figure 3a). The qRT-PCR experiments were performed after checking the similar amplification efficiency of the PCR reactions and RNA quality of all RNA samples. Furthermore, we observed a significant decrease in cell proliferation in LAT1-suppressed cells compared with control siRNA cells (*p* < 0.05, Figure 3b).

### 2.5. Association between mTOR and Oxaliplatin in LAT1-Suppressed CRC Cells

**Figure 4:**
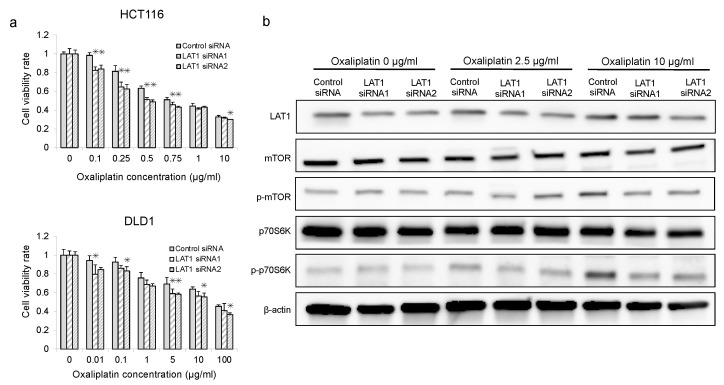
Analysis of oxaliplatin chemosensitivity and mTOR-related proteins in LAT1-suppressed CRC cells. (**a**) Oxaliplatin sensitivity was evaluated in HCT116 and DLD1 cells treated with LAT1 siRNAs using a Cell Counting Kit-8 kit. * *p* < 0.05, ** *p* < 0.01. (**b**) LAT1, mTOR, p-mTOR, p70S6K, and p-p70S6K expression in LAT1 suppressed CRC cells after treatment with oxaliplatin for 48 h, visualized using Western blot. β-Actin was used as the loading control.

LAT1-suppressed CRC cells exhibited significantly enhanced sensitivity to oxaliplatin compared to control cells (*p* < 0.05, Figure 4a). Therefore, to clarify the relationship between LAT1 and anti-cancer drug-induced mTOR activation, Western blotting was used to determine the protein expression of LAT1, mTOR, p-mTOR, p70S6K, and p-p70S6K in LAT1-suppressed CRC cells after treatment with oxaliplatin. Oxaliplatin induced the expression of p-mTOR and p-p70S6K in control cells, which was consistent with the findings of a previous report (Figure 4b) [15]. Interestingly, the levels of oxaliplatin-induced mTOR signal proteins (p-mTOR and p-p70S6K) were reduced in LAT1-suppressed cells compared to those in control siRNA cells (Figure 4b) (Supplementary Figure S2).

## 3. Discussion

This study demonstrated an association between high LAT1 expression and cancer aggressiveness, recurrence, and poor prognosis in a cohort of 98 CRC patients treated with adjuvant chemotherapy. We found that high LAT1 expression correlated with shorter recurrence-free survival and overall survival in patients treated with adjuvant CAPOX, but this correlation was not observed in the UFT + LV and capecitabine treatment groups. Multivariate analyses demonstrated that positive membrane LAT1 expression was an independent predictor of recurrence in CRC patients treated with the adjuvant CAPOX regimen. Furthermore, the proliferation potency and oxaliplatin resistance in LAT1-suppressed CRC cell lines were suppressed compared to control cells in vitro, and LAT1 suppression canceled oxaliplatin-induced accumulation of p-mTOR and p-p70S6K, which have been identified as chemoresistance-related signals.

Previous studies have examined the association between LAT1 expression and the response to adjuvant chemotherapy in pancreatic cancer [22] and gastric cancer [26], and revealed that patients with high LAT1 expression had shorter survival intervals. Consistent with these reports, we found that LAT1 knockdown increased oxaliplatin sensitivity and high membrane LAT1 expression was an independent predictor for poor survival and recurrence in CRC patients receiving an adjuvant CAPOX regimen, but not in our adjuvant UFT + LV and capecitabine groups. These observations suggest that pre-treatment evaluation of membrane LAT1 in curatively-resected CRC samples could be a marker to predict high-risk CRC cases with short survival rates, high recurrence rates, and poor response to adjuvant chemotherapy, including oxaliplatin.

The mTOR protein has been reported to be activated by increased amino acid uptake, and is associated with chemoresistance in several cancers [15,27,28,29,30]. Amino acid transporters, including LAT1, can activate mTOR signals and various cancer-related molecules (HIF1A, growth factors, and kinases) through regulation of amino acid metabolism and protein synthesis, leading to cancer stemness and metastatic invasiveness, as well as therapeutic resistance [31,32]. However, few studies have been conducted to clarify how the mTOR signal associated with therapeutic resistance in CRC is activated during exposure to anti-cancer drugs. In this study, we clarified that the amino acid transporter LAT1 is overexpressed in the cancerous region compared to the non-cancerous region, and that LAT1 suppression increased oxaliplatin sensitivity and inhibited the oxaliplatin-induced accumulation of p-mTOR and p-p70S6k. Moreover, we reported a positive correlation between LAT1 accumulation and mTOR signal activation in clinical cancer specimens [18,23].

These findings suggest that mTOR signaling during treatment might be activated by constitutive amino acid transporter LAT1 overexpression, regulating amino acid uptake and in response to oxaliplatin, at least in CRC patients. However, LAT1 and mTOR have been known to regulate cancer-related molecules not only tumor-side, but also host-side, acting on factors of the tumor microenvironment such as the extracellular matrix, stromal cells, endothelial cells, and immune cells [33]. Further studies are needed to analyze whether LAT1 targeting against the tumor microenvironment affects cancer-related molecules other than mTOR in patients with CRC during chemotherapy.

In this study, cell viability in LAT1-suppressed CRC cells was inhibited compared to controls, indicating the potential of LAT1 as a therapeutic target for CRC. Some LAT1 inhibitors have already been developed and have shown significant anti-cancer effects [14,34]. Moreover, LAT1 has been reported to be expressed in cancer stem cells that contribute to cancer aggressiveness and resistance to therapy, and LAT1 suppression inhibits the immune checkpoint protein programmed cell death-1 ligand 1, a clinically applied target, in cancer stem cells. These findings suggested that LAT1 targeting strategies against cancer cells are promising [35]. On the other hand, it has been reported that enhanced amino acid uptake by LAT1 is critical for human T-cell activation, and that suppression of LAT on immune cells may avoid excessive immune responses [33]. This report, and recent findings on tumor immunity, suggest that systemic LAT1 inhibition in cancer patients is beneficial in terms of its anti-cancer effect by suppressing mTOR signaling, programmed cell death-1 ligand 1, expression, and cancer stemness, but is not beneficial through suppression of activated T-cells, which require increased amino acid uptake via LAT1 to maintain the T-cell activation state. In other words, systemic LAT1 inhibition may potentially be tumor-promoting in terms of host-side immunity [36,37]. Therefore, to develop therapeutic tools to inhibit LAT1, more tumor-specific drug delivery methods (e.g., drug-conjugated antibodies, liposome-encapsulated drugs, and improvement of the tumor microenvironment) should be utilized for future clinical applications.

This study had several limitations. First, the CRC patient cohort that underwent radical resection and adjuvant treatment was small. Second, the CRC patients were not consecutive, because mostly advanced patients undergoing radical resection with adjuvant therapy were retrospectively selected. Therefore, our data may not be generalizable to all patients with CRC, including those with unresectable tumors. In the future, a large-cohort prospective evaluation of LAT1 using pre-treatment surgical specimens or biopsy tissues is warranted, in order to establish the significance of LAT1 evaluation in whole CRC cases with and without adjuvant chemotherapy.

## 4. Materials and Methods

### 4.1. Clinical Samples and Cell Lines

Surgical specimens were obtained from 98 patients with CRC (59 men and 39 women) who underwent potentially curative surgery and post-operative adjuvant chemotherapy, including 43 patients undergoing the capecitabine-plus-oxaliplatin (CAPOX) regimen, 35 patients who received oral uracil-tegafur plus leucovorin (UFT + LV), 14 patients who were administered oral capecitabine, and others (*n* = 6) at the Department of General Surgical Science, Gunma University (Maebashi, Gunma, Japan), between 2013 and 2016. None of the patients had received preoperative radiation or chemotherapy. This study conformed to the tenets of the Declaration of Helsinki, and was approved by the Institutional Review Board for Clinical Research of Gunma University Hospital (approval number: HS2020-102). Patient consent was obtained using the opt-out method. Radical surgical resection was defined as a case lacking evidence of residual tumor and microscopic tumor-free resection margins. All sample data, including age, sex, history of smoking, tumor factor, N-factor, tumor location, pathological stage, histological type, lymphatic invasion, vascular invasion, perineural invasion, recurrence, survival time, and adjuvant chemotherapy regimens were obtained from clinical and pathologic records.

Human CRC cell lines, HCT116 and DLD1, were purchased from the American Type Culture Collection. They were cultured in Dulbecco’s modified eagle medium (Wako, Osaka, Japan) supplemented with 10% fetal bovine serum and 1% penicillin-streptomycin. Cultured cells were incubated in a humidified atmosphere containing 5% carbon dioxide at 37 °C.

### 4.2. Immunohistochemical Analysis

Paraffin-embedded CRC specimens were cut into 4 µm thick sections and mounted on glass slides. All sections were incubated at 60 °C for 60 min, deparaffinized in xylene, rehydrated, and incubated with fresh 0.3% hydrogen peroxide in 100% methanol for 30 min at room temperature to block endogenous peroxidase activity. After rehydration through a graded series of ethanol treatments, antigen retrieval was performed using an Immunosaver (Nishin EM, Tokyo, Japan) at 98 °C–100 °C for 45 min. The sections were passively cooled to room temperature and incubated in Protein Block Serum-Free Reagent (DAKO, USA) for 30 min. The specimens were then incubated with mouse monoclonal LAT1 antibody (Kyowa Hakko; dilution of 1:800) in Dako REAL Antibody Diluent at 4℃ for 24 h. Primary antibody staining was visualized using the Histofine Simple Stain MAX-PO (Multi) Kit (Nichirei, Tokyo, Japan), according to the manufacturer’s instructions. Chromogen 3,3-diaminobenzidine tetrahydrochloride was applied as a 0.02% solution in a 50 mM ammonium acetate-citrate acid buffer (pH 6.0) containing 0.005% hydrogen peroxide. The sections were lightly counterstained with hematoxylin and mounted. Negative controls were incubated without the primary antibody, and no detectable staining was evident. Immunohistochemical slides were evaluated by three experienced researchers who were blinded to the clinical data. The staining score for each sample was set as the average of the evaluations of the three researchers. Immunohistochemistry was performed based on the percentage of membrane LAT1 staining. A staining area of >10% was defined as the positive group, and ≤10% was defined as the negative group.

### 4.3. siRNA Transfection

LAT1-specific siRNA oligonucleotides (LAT1 siRNA1, GGAACAUUGUGCUGGCAUUtt; LAT1 siRNA2, GUGUGAUGACGCUGCUCUAtt) and non-targeting control siRNA oligos (control siRNA) were purchased from Dharmacon. The target cell lines, HCT116 and DLD1, were subjected to RNA interference using an in vitro electroporation protocol. Briefly, cells were suspended in serum-free Opti-MEM I (Life Technologies) at a density of 1 × 10^7^ cells/mL, after which siRNA was added to the cell suspension at a concentration of 3 µM. Next, 100 μL of the cell suspension was transferred to a 2 mm gap cuvette electrode and subjected to electroporation using an electroporator (CUY21EDIT II; BEX Co., Japan).

### 4.4. Protein Extraction and Western Blot Analysis

Total protein (10 μg) was electrophoresed on a polyacrylamide gel and electroblotted onto a nitrocellulose membrane. Western blotting was performed to confirm the expression of target proteins using the following reagents: anti-LAT mouse monoclonal antibody (1:800; Kyowa Hakko), anti-mTOR rabbit polyclonal antibody (1:1000; Cell Signaling Technology, #2972), anti-p-mTOR rabbit polyclonal antibody (1:1000; Cell Signaling Technology, #2971), anti-p70S6K rabbit monoclonal antibody (1:1000; Cell Signaling Technology, #2708), anti-p-p70S6K rabbit polyclonal antibody (1:1000; Cell Signaling Technology, #9205), and β-actin mouse monoclonal antibody (1:1000; Sigma-Aldrich). Expression of β-Actin was used as a protein loading control. The blots were detected using an enhanced chemiluminescence Western Blot Analysis Detection System and an Image Quant LAS 4000 machine (GE Healthcare Life Sciences).

### 4.5. Reverse Transcription Polymerase Chain Reaction (RT-PCR)

Quantitative real-time RT-PCR analysis was performed to determine the expression of LAT1 mRNA in HCT116 and DLD1 CRC cell lines treated with siRNAs. These cells were kept at −80 °C until RNA extraction. Total RNA was extracted from CRC cell lines using an RNeasy Kit (Qiagen, Hilden, Germany), and the quantity and quality of total RNA were measured using an ND-1000 spectrophotometer (NanoDrop Technologies, Wilmington, DE, USA) and Bioanalyzer 2100 (Agilent). The RNA Integrity Number (RIN) of all total RNA samples for qRT-PCR was more than 9.0 (RIN data of HCT116: Control siRNA, 9.80; LAT1 siRNA1, 9.80; LAT1 siRNA2 9.90. RIN data of DLD1: Control siRNA, 9.60; LAT1 siRNA1, 9.60; LAT1 siRNA2 9.60.). Specific oligonucleotide primers of LAT1 (Accession number: AB018009) were designed to amplify a 103-bp PCR product. The following primers were used: LAT1 sense primer, 5′ -ATCGGGAAGGGTGATGTGTCCAAT-3′and antisense primer, 5′-CAAAGAGGCCGCTGTATAATGCCA-3′; β-actin sense primer, 5′-CCAACCGCGAGAAGATGA-3′ and antisense primer, 5′-CCAGAGGCGTACAGGGATAG-3′; GAPDH sense primer, 5′-CTCTGCTCCTCCTGTTCGAC-3′ and antisense primer, 5′-GCCCAATACGACCAAATCC-3′. β-actin and GAPDH were used as reference genes to normalize the RNA input for all RT-PCR analyses and to evaluate the suppression levels of LAT1 by specific siRNA treatment between siRNA-treated identical CRC cell lines. PCR amplification of LAT1, β-actin, and GAPDH mRNA in cell lines was performed in the Light Cycler^®^ 480 system (Roche Applied Science, IN, USA) using the GoTaq 1- Step RT-qPCR System (Promega, Madison, WI, USA) according to the manufacturer’s protocol. As both reference genes showed the same tendency to suppress LAT1, the results using β-actin were used as the main data. In brief, a master mixture for one PCR reaction was prepared on ice, containing one μL of 100 ng total RNA, ten μL of GoTaq qPCR Master mix (2×), 0.4 μL GoScript RT Mix for 1-Step RT-qPCR (50×), 0.04 μL of 50 μM primers (final concentration: 250 nM), and 0.8 μL of 25 mM MgCl_2_ (final concentration: 25 mM). The final volume was adjusted to 20 μL with nuclease-free water. The reaction mixture was exposed to the following cycling conditions: reverse transcription at 37 °C for 15 min; RT inactivation and hot-start activation at 95 °C for 10 min; PCR, 40 cycles at 95 °C for 10 s, 62 °C for 30 s, and 72 °C for 30 s. The amplified PCR products were subjected to a temperature gradient from 60 °C to 95 °C at 0.2 °C/second under continuous fluorescence monitoring to produce a melting curve of the products. The relative expression levels of candidate genes were calculated using the 2^−ΔΔCT^ method after checking the similar amplification efficiency (LAT1, 1.983; β-actin, 1.965; GAPDH, 2.030). Only one peak for each sample was observed.

### 4.6. Cell Proliferation Assay

Proliferation analysis of CRC cell lines treated with control or LAT1 siRNA was performed. The cells were seeded in 96-well plates (approximately 2000 cells per well in 100 μL of medium containing 10% FBS). After 0, 24, 48, 72, and 96 h, cell proliferation was measured using a Cell Counting Kit-8 (Dojindo Laboratories, Tokyo, Japan). Ten microliters of the cell counting solution were added to each well and incubated for 2 h at 37 °C. The absorbance of each well was measured using a microplate reader (Thermo, Waltham, MA, USA) at 450 nm.

### 4.7. Oxaliplatin Sensitivity Assay

The oxaliplatin sensitivity of cells treated with control or LAT1 siRNA was measured. Cells were plated in 96-well plates in 100  µL of the medium at 2000 cells/well density. After 24 h incubation, the cells were treated with various concentrations of oxaliplatin (Pfizer) (HCT116:0, 0.1, 0.25, 0.5, 0.75, 1, and 10 μg/mL) (DLD1:0, 0.01, 0.1, 1.0, 5.0, 10, and 100 μg/mL) for 48 h. Cell viability was assessed using Cell Counting Kit-8 (10 μL per well for 2 h at 37 °C) and by measuring the absorbance of the medium at 450 nm using a microtiter plate reader (Thermo, Waltham, MA, USA). Protein extraction and Western blotting of CRC cell lines treated with control or LAT1 siRNA were performed after oxaliplatin treatment (0, 2.5, and 10 μg/mL) for 72 h.

### 4.8. Statistical Analysis

The χ^2^ test was used to identify statistically significant differences between the groups. Differences between the three groups were evaluated using analysis of variance with Tukey’s multiple comparison test. Kaplan–Meier curves of overall survival and recurrence-free survival were generated from clinical data. Statistical significance was determined using the log-rank test. Univariate and multivariate analyses for overall and recurrence-free survival were performed using the Cox proportional hazard model. In addition, the relative multivariate significance of potential prognostic factors based on the significant prognostic value in univariate analysis was investigated to test the independent prognostic contribution of membrane LAT1 expression. A *p*-value of <0.05 was considered statistically significant. All statistical analyses were performed using JMP Pro 15.2.0 software (SAS Institute, Cary, NC, USA).

## 5. Conclusions

We observed a correlation between high LAT1 expression levels and shorter overall survival and recurrence-free survival durations in CRC patients treated with oxaliplatin-based adjuvant chemotherapy. LAT1 evaluation before adjuvant treatment may be a sensitive marker for oxaliplatin-based regimens. Moreover, our in vitro analysis further clarified that LAT1 suppression in CRC cells increased oxaliplatin sensitivity via suppression of oxaliplatin-induced p-mTOR and p-p70S6K. Therefore, LAT1 targeting may be promising for preventing recurrence after adjuvant chemotherapy and treatment in patients with refractory CRC.

## Figures and Tables

**Table 1:** Relationship between LAT1 positivity and various clinicopathological factors.

Variable	Membrane LAT1 Expression		*p*-Value
	Negative (*n* = 45)	Positive (*n* = 53)	
Age			
70≧	35	36	0.274
70<	10	17	
Sex			
Male	26	33	0.651
Female	19	20	
Smoking			
Yes	23	28	0.865
No	22	25	
Tumor factor			
T1–T3	36	40	0.591
T4	9	13	
N factor			
Absent	17	9	0.020 *
Present	28	44	
Tumor location			
Right	12	18	0.434
Left	33	35	
Pathological stage			
Ⅱ	17	11	0.063
Ⅲ	28	42	
Histology Type			
Well	26	35	0.233
Not-well	19	18	
Lymphatic invasion			
Absent	11	5	0.044 *
Present	34	48	
Vascular invasion			
Absent	10	17	0.274
Present	35	36	
Perineural invasion			
Absent	26	30	0.091
Present	19	23	
Recurrence			
Absent	41	37	0.007 *
Present	4	16	
Adjuvant chemotherapy			
CAPOX	21	22	0.627
UFT + LV	14	21	
Capecitabine	6	8	
Others	4	2	

* *p <* 0.05.

**Table 2:** Univariate and multivariate analysis of clinicopathological factors affecting recurrence-free survival rate following surgery in 98 CRC patients.

Factors		Univariate Analysis			Multivariate Analysis		
		Hazard Ratio	95% CI	*p*-Value	Hazard Ratio	95% CI	*p*-Value
Age	70≥	Reference					
	70<	1.241	0.477–3.232	0.658			
Sex	Male	Reference					
	Female	0.477	0.173–1.314	0.152			
Smoking	Yes	Reference					
	No	0.695	0.284–1.702	0.426			
T factor	T1–3	Reference			Reference		
	T4	3.808	1.583–9.161	0.003 *	3.282	1.309–8.234	0.011 *
N factor	Absent	Reference					
	Present	3.493	0.810–15.06	0.093			
Tumor location	Right	Reference					
	Left	0.803	0.320–2.014	0.640			
Pathological stage	Ⅱ	Reference					
	Ⅲ	2.356	0.690–8.040	0.171			
Histology	Well	Reference					
	Not-well	0.87	0.347–2.182	0.767			
Lymphatic invasion	Absent	Reference					
	Present	1.93	0.448–8.333	0.377			
Vascular invasion	Absent	Reference					
	Present	4.11	0.953–17.75	0.058			
Perineural invasion	Absent	Reference			Reference		
	Present	2.661	1.061–6.674	0.037 *	1.91	0.729–4.994	0.189
Adjuvant chemotherapy	UFT + LV	Reference					
	CAPOX	2.42	0.770–7.601	0.13			
	Capecitabine	1.301	0.238–7.107	0.761			
LAT1	Negative	Reference			Reference		
	Positive	4.003	1.337–11.99	0.013 *	3.99	1.327–11.99	0.014 *

CI, confidence interval; * *p* < 0.05.

## Data Availability

The data presented in this study are available on request from the corresponding author.

## Supplementary Materials

The following supporting information can be downloaded at: https://www.mdpi.com/article/10.3390/ijms24032604/s1, Figure S1: Kaplan–Meier survival curves of patients with CRC treated by UFT/LV or capecitabine according to LAT1 expression, Figure S2: Western blot analysis of LAT1, mTOR, p-mTOR, p70S6K, and p-p70S6K in CRC cell lines treated with LAT1 siRNA and oxaliplatin, Table S1: Univariate and multivariate analysis of clinicopathological factors affecting recurrence-free survival rate following surgery in 43 CRC patients treated by adjuvant CAPOX.
