# Supplementary material for: Association of High LAT1 Expression with Poor Prognosis and Recurrence in Colorectal Cancer Patients Treated with Oxaliplatin-Based Adjuvant Chemotherapy
Source: Int J Mol Sci. 2023 Jan 30;24(3):2604. doi: 10.3390/ijms24032604 (PMC9916902; doi:10.3390/ijms24032604)
Supplement: Supplementary file 1 [file ijms-24-02604-s001.zip › ijms-2082446-supplementary.pdf]

Supplementary material

Association of High LAT1 Expression with Poor Prognosis and Recurrence in Colorectal Cancer Patients Treated with Oxaliplatin-Based Adjuvant Chemotherapy

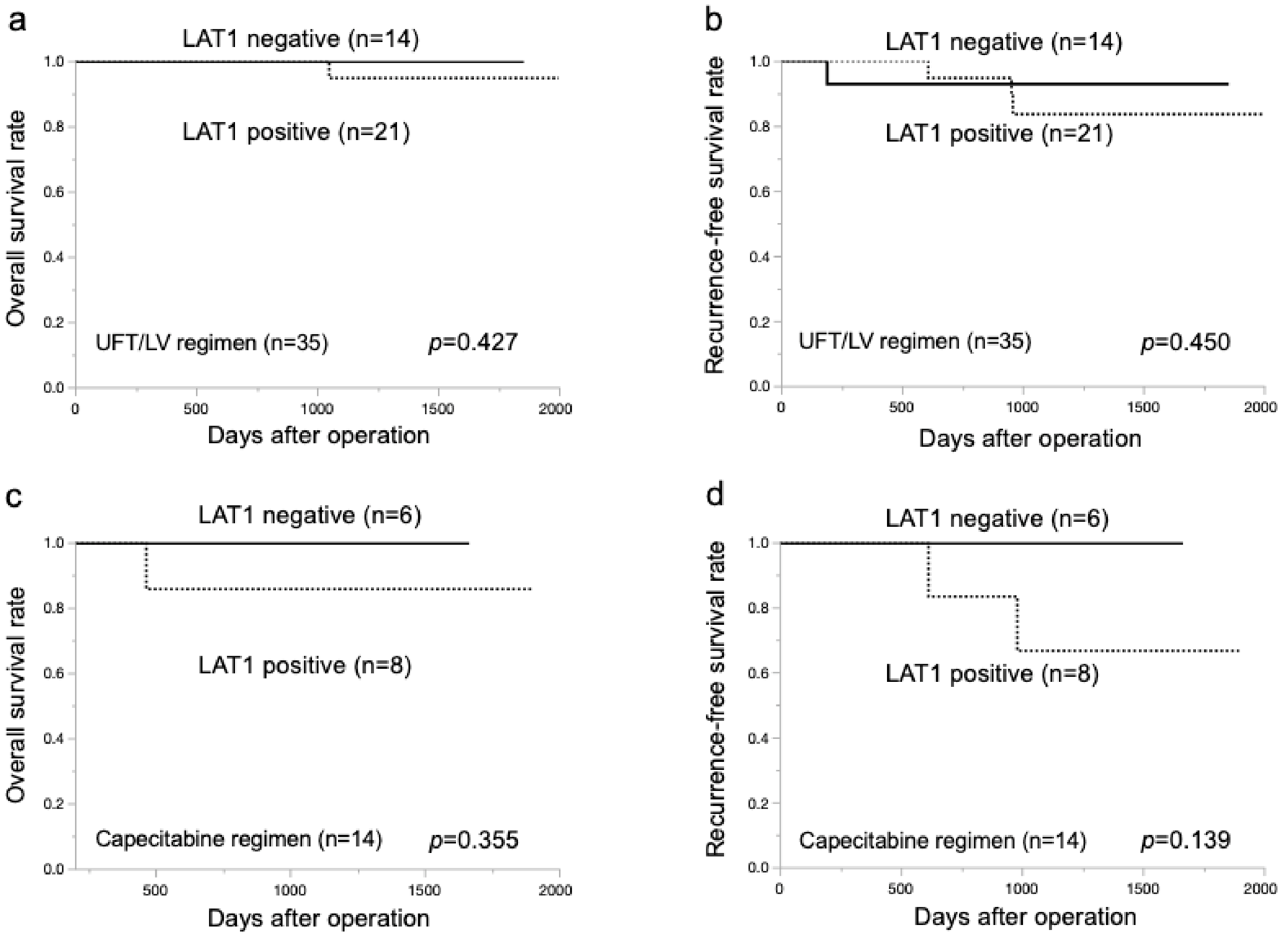

Figure S1. Kaplan–Meier survival curves of patients with CRC treated by UFT/LV or capecitabine according to LAT1 expression (**a**) (**b**) Kaplan–Meier analysis of overall survival and recurrence-free survival in 35 CRC patients treated by UFT/LV regimen according to LAT1 expression ($p = 0.4267$ and $p = 0.45$, respectively). (**c**) (**d**) Kaplan–Meier analyses of overall survival and recurrence-free survival in 14 CRC patients treated with capecitabine according to LAT1 expression ($p = 0.355$ and $p = 0.139$, respectively).

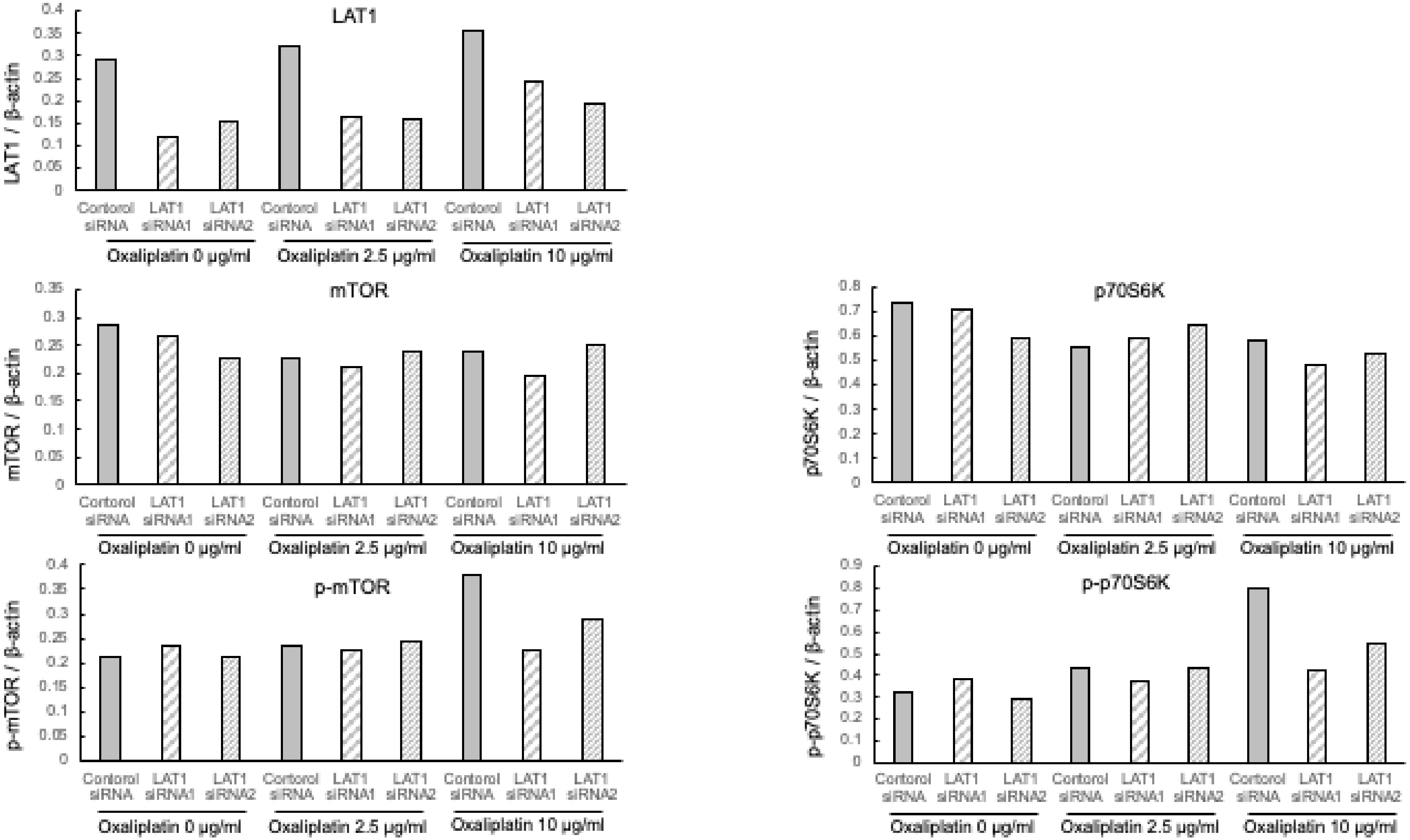

Figure S2. Western blot analysis of LAT1, mTOR, p-mTOR, p70S6K, and p-p70S6K in CRC cell lines treated with LAT1 siRNA and oxaliplatin These proteins were normalized to the level of β-actin expression. The Intensity of their blots was measured using ImageJ software (Rasband, W.S., ImageJ, U. S. National Institutes of Health, Bethesda, Maryland, USA).

Table S1. Univariate and multivariate analysis of clinicopathological factors affecting recurrence-free survival rate following surgery in 43 CRC patients treated by adjuvant CAPOX.

Factors		Univariate Analysis			Multivariate Analysis		
		Hazard ratio	95% CI	*p*-value	Hazard ratio	95% CI	*p*-value
Age	70≥		Reference			Reference	
	70<	3.853	1.102-13.48	0.035*	2.054	0.391-10.80	0.395
Sex	Male		Reference				
	Female	0.934	0.273-3.191	0.913			
Smoking	Yes		Reference				
	No	0.862	0.263-2.825	0.806			
T factor	T1-3		Reference				
	T4	3.191	0.973-10.46	0.056			
N factor	Absent		Reference				
	Present	640866872	0- ▪	0.999			
Tumor location	Right		Reference			Reference	
	Left	0.299	0.091-0.987	0.048*	0.508	0.102-2.527	0.408
Pathological stage	Ⅱ		Reference				
	Ⅲ	1.84	0.234-14.45	0.562			
Histology	Well		Reference				
	Not-well	0.58	0.169-1.972	0.38			
Lymphatic invasion	Absent		Reference				
	Present	599081383	0- ▪	0.999			
Vascular invasion	Absent		Reference				
	Present	692416517	0- ▪	0.999			
Perineural invasion	Absent		Reference				
	Present	2.324	0.680-7.946	0.179			
LAT1	Negative		Reference			Reference	
	Positive	13.32	1.685-105.2	0.014*	12.53	1.549-101.4	0.018*

CI, confidence interval; *$p<0.05$.
